# Reproducibility of Neonate Ocular Circulation Measurements Using Laser Speckle Flowgraphy

**DOI:** 10.1155/2015/693056

**Published:** 2015-10-18

**Authors:** Tadashi Matsumoto, Takashi Itokawa, Tomoaki Shiba, Yuji Katayama, Tetsushi Arimura, Norio Mizukaki, Hitoshi Yoda, Yuichi Hori

**Affiliations:** ^1^Department of Ophthalmology, Toho University Omori Medical Center, 6-11-1 Omori-nishi, Ota-ku, Tokyo 143-8541, Japan; ^2^Department of Neonatology, Toho University Omori Medical Center, 6-11-1 Omori-nishi, Ota-ku, Tokyo 143-8541, Japan

## Abstract

Measuring the ocular blood flow in neonates may clarify the relationships between eye diseases and ocular circulation abnormalities. However, no method for noninvasively measuring ocular circulation in neonates is established. We used laser speckle flowgraphy (LSFG) modified for neonates to measure their ocular circulation and investigated whether this method is reproducible. During their normal sleep, we studied 16 subjects (adjusted age of 34–48 weeks) whose blood flow could be measured three consecutive times. While the subjects slept in the supine position, three mean blur rate (MBR) values of the optic nerve head (ONH) were obtained: the MBR-A (mean of all values), MBR-V (vessel mean), and MBR-T (tissue mean), and nine blood flow pulse waveform parameters in the ONH were examined. We analyzed the coefficient of variation (COV) and the intraclass correlation coefficient (ICC) for each parameter. The COVs of the MBR values were all ≤10%. The ICCs of the MBR values were all >0.8. Good COVs were observed for the blowout score, blowout time, rising rate, falling rate, and acceleration time index. Although the measurement of ocular circulation in the neonates was difficult, our results exhibited reproducibility, suggesting that this method could be used in clinical research.

## 1. Introduction

Laser speckle flowgraphy (LSFG) can be used to noninvasively measure ocular blood flow [1–4]. One indicator of ocular blood flow is the mean blur rate (MBR) [5]. LSFG is used for research on various diseases in adult patients, such as glaucoma [5, 6], retinal vein occlusion [7], and diabetic retinopathy [8]. It is also used in research related to systemic diseases such as diabetes and chronic kidney disease [9], as well as studies on the link between aging and retinal blood flow changes [10–12]. However, no noninvasive method to measure ocular circulation has been established for neonates.

Other than LSFG, the currently available devices that allow us to noninvasively measure ocular blood flow include Doppler FD-optical coherence tomography (OCT) [13] and scanning laser Doppler flowmetry (SLDF) [14]. Although Doppler FD-OCT and SLDF can be used to measure the absolute values of blood flow speed, in cases of structurally poor fixation it is difficult not only to measure the values but also to downsize the devices. Because LSFG does not offer absolute values for the blood flow rate, it is challenging to make comparisons between individuals. However, downsizing LSFG is possible due to the simplicity of the system, and it also provides a wide image field of view.

Ocular diseases in neonates such as the retinopathy of prematurity (ROP) changes in retinal circulation occur with dilation and tortuosity of retinal blood vessels [15, 16]. We believe that by noninvasively measuring and researching retinal hemodynamics in neonates the relationships between neonatal ocular diseases and ocular circulation abnormalities could be clarified.

Here we measured the ocular blood flow in neonates using a version of LSFG modified for use in neonate patients, and we assessed the reproducibility of this method to determine whether the results obtained by this method can be used clinically.

## 2. Subjects and Methods

### 2.1. Subjects

The subjects for our investigation of reproducibility were nonincubator neonates who were not on respirators. For each subject, an ophthalmologist had requested an examination due to suspicion of ROP or other diseases, between October 2014 and April 2015 at the Toho University Omori Medical Center. Including the cases of the same neonate undergoing testing at multiple weeks, we attempted testing in 55 neonates during their normal sleep, and we determined the test success rate. We studied 16 neonates (16 eyes) during sleep in whom the circulation could be measured three times consecutively at the initial test (male : female ratio, 9 : 7; adjusted age, 34–48 weeks).

The presence/absence of ocular diseases and systemic diseases was investigated. With regard to eye diseases, there were 14 neonates with no ROP and two neonates with ROP. No severe systemic diseases requiring treatment were noted in the subjects.

This study was conducted in accord with the principles laid out in the Declaration of Helsinki, and the data analysis was approved by the ethical review committee of Toho University (number 26-96).

### 2.2. LSFG Measurement

**Figure 1:**
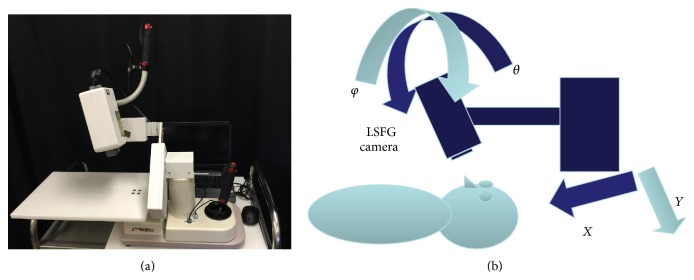
(a) LSFG-baby. Examination of neonates in the supine position. (b) Operability of the LSFG-baby system. The system's camera is set on a tilting stage with two axes (*φ* and *θ*) and *x*-*y* stages to adjust the field of view.

Measurements were obtained using the “LSFG-baby” system, which is a version of the commercially available LSFG-NAVI (Softcare, Fukuoka, Japan) modified in such a manner that the testing can be performed with the neonate in the supine position (Figure 1). The camera provided with the LSFG-baby system is set on a tilting stage with two axes (*φ* and *θ*) and *x*-*y* stages to adjust the field of view.

For the testing, pupillary dilation was achieved with 2.625% phenylephrine hydrochloride, 0.125% tropicamide, and 0.25% cyclopentolate, after which the ONH was imaged for 3 sec. Testing was performed while the infant was sleeping to measure his or her circulation during rest. All measurements were performed by the same tester. Testing was concluded within 10 min, and if it was difficult to measure both eyes within this time, only the left eye was measured. After testing, the images were confirmed, and results that were significantly out of focus or for which only two heartbeats or less were measured were excluded.

### 2.3. Analysis of Reproducibility

To evaluate the reproducibility of the measurement method, the three MBR parameters of MBR-A (mean of all values), MBR-V (vessel mean), and MBR-T (tissue mean) were measured in the ONH [17] using the LSFG Analyzer software (Softcare).

We performed a pulse waveform analysis of the ONH blood flow, and nine pulse waveform parameters [17] were calculated: fluctuation, skew, blowout score (BOS), blowout time (BOT), rising rate, falling rate, flow acceleration index (FAI), acceleration time index (ATI), and resistivity index (RI). The analysis was performed using the ONH MBR and pulse waveform parameters from the LSFG-baby system with which the coefficient of variation (COV) and intraclass correlation coefficient (ICC) were measured three consecutive times.

### 2.4. Statistical Analysis

The data were analyzed using the 11.2 version of the statistical software JMP (SAS, Cary, NC).

## 3. Results

**Figure 2:**
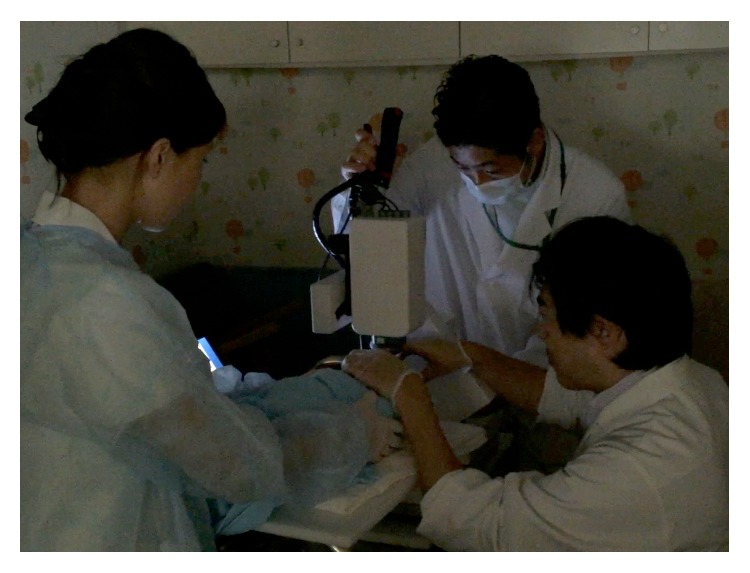
Three physicians oversaw the infant's fixation, opening of eyelids, and imaging.

**Figure 3:**
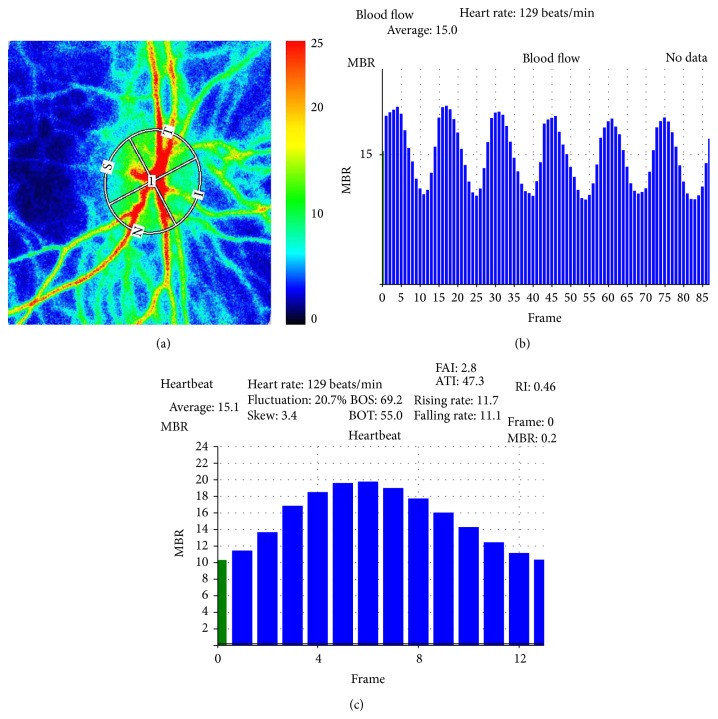
(a) In the color-coded maps, red indicates high blood flow and blue indicates low blood flow. The image is observed indirectly. (b) Blood flow. MBR of 3 s and 90 frames (30/s). (c) Heartbeat. Average MBR per heartbeat.

We were able to measure neonate ONH blood flows by using the LSFG-baby system (Figure 2). Three physicians oversaw the infant's fixation, opening of eyelids, and imaging. We were also able to analyze pulse waveforms by setting a rubber band on the ONH (Figure 3). In our testing of all 55 cases, we defined “test success” as the case when the measurement was successfully conducted with quality that was high enough for at least one pulse waveform analysis to be performed.

Based on this definition, we determined the success rates. The test success rate for LSFG-baby modified for neonate use was 72.7% during normal sleep (40/55 cases). The reasons for test failure were as follows: (1) awakening during sleep in 5 of 15 cases; (2) not being able to conduct the measurement because of strong ocular movements during sleep in 9 cases; and (3) the measurement being impossible because of the eyeball being fixated upward. The subjects who were awake from the beginning were not included in the number of cases.

We found that it was not possible to perform the test on neonates who were awake from the start of testing. Even among the sleeping neonates, it was difficult or impossible to perform the test with an infant who exhibited strong ocular movements or had off-center vision fixation.

Regarding the reproducibility of the testing, Table 1 shows the results of the 16subjects for which measurements could be obtained three times. Because it was difficult to measure the IOP in neonates, we did not measure the IOP in this study.

Table 2 shows the COVs and ICCs for the ONH MBR values and pulse waveform parameters in the 16 subjects. The COVs of the MBR values were all ≤10%, and the ICCs of the MBR values were all >0.8. Good results were also achieved for the pulse waveform parameter COVs at ≤10% for the BOS, BOT, rising rate, falling rate, and ATI. The COV results for fluctuation, skew, FAI, and RI exceeded 15%.

The pulse waveform parameter ICCs were >0.6 for fluctuation and FAI. However, the ICCs for the skew, BOS, BOT, rising rate, falling rate, ATI, and RI were all ≤0.4.

LSFG offers International Electrotechnical Commission global standard class 1 safety and is believed to be safe for use on neonates [1–3]. Indeed, no adverse events occurred during our study.

## 4. Discussion

In this study, the test success rate was 72.7% during normal sleep. The COVs of the MBR values were all ≤10%, and the COVs were good for the BOS, BOT, rising rate, falling rate, and ATI. In addition, the ICCs of the MBR values were all >0.8.

Although contrast-enhanced tests such as fluorescein angiography (FA) are useful for ocular circulation measurement [18, 19], the use of a contrast medium makes it difficult to call these tests noninvasive, and repeated testing is likely to be difficult. Noninvasive circulation testing methods include color Doppler imaging (CDI) with neonates.

Holland et al. [20] and Neely et al. [21] used CDI to measure blood flow in the retinal central artery and ophthalmic artery in ROP patients and found that it was difficult to accurately measure the retinal blood flow by this method. Because LSFG can measure retinal hemodynamics over an extensive range in real time [3], we felt that testing would be possible even for neonates, who have poor fixation, if there was only a certain amount of movement.

The clinical application of blood flow observations using LSFG requires ease of operability and reproducibility of results. Compared to adults, neonates exhibit a large amount of body and ocular movement and have poor fixation, making testing difficult. Nevertheless, the one-time test success rate in the present study was 72.7% during normal sleep. Although this does not reach the 94% success rate for retinal central artery blood flow measurement using CDI [20], we believe that the method of testing that we used offers a certain level of operability and an acceptable success rate.

A high proportion of neonate sleep is rapid eye movement (REM) sleep [22], and eye movement is often observed while neonates are sleeping. Eye movement is still observed when sedatives are used, unlike during general anesthesia. In the present study, the causes of test failure were related to awakening and ocular movement, but since general anesthesia is invasive, we do not believe that it could be used for every test.

Our reproducibility evaluation revealed good COVs for the ONH MBR and pulse waveform parameters of BOS, BOT, rising rate, falling rate, and ATI, but the COVs fluctuation, skew, FAI, and RI were poor. As Tsuda et al. reported [12], fluctuation, skew, and FAI respond sensitively to slight changes in blood flow waveforms. For neonates, who exhibit a heart rate nearly double that of adults, the number of frames during one heartbeat is approximately one-half that of adults. Therefore, changes in one frame more significantly affect the fluctuation, skew, and FAI in neonates than in adults. Moreover, RI may also have exhibited greater fluctuations than in adults because neonates have lower minimum blood flow values.

The ICCs of the BOS, BOT, rising rate, falling rate, and ATI were poor, but the COVs were good. An ICC becomes low when there is little individual difference, and this may be related to the present ICC results. We think that the reproducibility of waveform parameters requires further investigation.

As there have been no reports of ocular circulation measurements in neonates using LSFG, a simple comparison cannot be made; however, studies using LSFG-NAVI for adults have indicated COVs of 0.9%–3.8% and ICCs of 0.95 to 0.98 [5]. Studies using LSFG for patients in the supine position during surgery have indicated COVs of 3.1% to 6.9% [23] and 6.7% for awake patients in the supine position [24]. In the present study, the COVs ranged from 7.7% to 9.7%, suggesting that the reproducibility for neonates was lower than for adults. Nevertheless, because the COV was ≤10% and ICC was >0.8 for the ONH MBR, we believe that the reproducibility of our method was of a level that would allow for clinical application.

The properties of LSFG also mean that it is difficult to use LSFG for neonates who are highly premature and have strong clouding of the cornea, crystalline lens, and/or vitreous body. In cases such as these, the MBR values results are likely to be inaccurate, and it would be difficult to investigate the extent of changes over time. Although it is thought that pulse waveforms would not be as significantly affected as blood flow values, in neonates, the small number of frames per heartbeat could make a waveform analysis inferior to that of adult cases.

In the future, if LSFG camera improvements result in an increase in the number of frames captured per heartbeat, more noninvasive and precise testing could be performed.

In the present study, the ONH MBR obtained from the neonates during normal sleep demonstrated good reproducibility. Therefore, we believe that the data obtained in this manner could be used as test data when performing clinical research.

In conclusion, the results of our study indicate that the measurement of ocular circulation in neonates using LSFG could contribute to clarifying the relationships between the ocular circulation and ocular diseases in neonates.

## Figures and Tables

**Table 1:** The neonates' demographic data.

	Normal sleep (*n* = 16)
Age (wks)	38.8 ± 3.6
Gestational age (wks)	33.0 ± 3.6
Weight (g)	2314.1 ± 398.2
Birth weight (g)	1867.5 ± 633.0
Heart rate/min	143.4 ± 10.1
Mean BP (mmHg)	48.2 ± 4.3
Gender (m : f)	9 : 7
ROP− : ROP+	14 : 2
Trisomy 21	3 cases
Mild VSD or ASD	4 cases

**Table 2:** Coefficients of variation and intraclass correlation coefficient for ONH blood flow values and waveform parameters.

	COVs (*n* = 16)	ICCs (*n* = 16)	Mean ± SD
MBR-A	7.7 ± 3.9	0.88	12.1 ± 3.5
MBR-V	9.2 ± 5.2	0.83	22.6 ± 6.7
MBR-T	9.7 ± 4.9	0.85	8.7 ± 2.7
Fluctuation	17.0 ± 8.6	0.75	17.2 ± 3.7
Skew	215.7 ± 500.1	0.15	4.7 ± 2.7
BOS	6.1 ± 3.9	0.37	72.1 ± 5.5
BOT	9.0 ± 7.3	0.17	56.2 ± 4.8
Rising rate	6.3 ± 5.2	0.14	11.8 ± 0.7
Falling rate	8.8 ± 6.5	0.05	11.4 ± 0.9
FAI	16.3 ± 10.1	0.65	2.4 ± 0.8
ATI	10.0 ± 7.4	0.15	44.8 ± 5.3
RI	15.0 ± 7.9	0.38	0.39 ± 0.07

MBR-A (all the mean), MBR-V (vessel mean), and MBR-T (tissue mean). BOS, blowout score; BOT, blowout time; FAI, flow acceleration index; ATI, acceleration time index; RI, resistivity index.
